# Correlation of retinal and choroidal microvascular impairment in systemic sclerosis

**DOI:** 10.1186/s13023-020-01649-5

**Published:** 2021-01-13

**Authors:** Felix Rommel, David Prangel, Michelle Prasuhn, Salvatore Grisanti, Mahdy Ranjbar

**Affiliations:** 1 Department of Ophthalmology, University of Lübeck, Ratzeburger Allee 160, 23562 Lübeck, Germany; 2 Laboratory for Angiogenesis and Ocular Cell Transplantation, University of Lübeck, Ratzeburger Allee 160, 23562 Lübeck, Germany

**Keywords:** Optical coherence tomography angiography, Systemic sclerosis, Retina, Choroid, Biomarker

## Abstract

**Purpose:**

To investigate the correlation between retinal and choroidal microperfusion in patients with systemic sclerosis (SSc) using optical coherence tomography angiography (OCTA).

**Methods:**

In this cross-sectional study SSc patients without clinical evidence of ocular involvement and healthy, age- and sex-matched volunteers were recruited. Participants underwent specific rheumatological and ophthalmological examinations, including optical coherence tomography (OCT) and OCTA. Retinal and choroidal thicknesses as well as perfusion of the retina and the choroidal sublayers were evaluated.

**Results:**

A total of 15 SSc patients (30 eyes) with a median disease duration of 60 months and 15 matched, healthy controls (30 eyes) were recruited. OCT data revealed a significantly lower macular volume, as well as Sattler’s layer and Haller’s layer thickness in SSc patients compared to controls. In OCTA analysis, the perfusion of both retinal plexus as well as Sattler’s and Haller’s layer were significantly reduced in the SSc group. Patients with a disease duration of more than 60 months showed a statistically significant positive correlation between retinal and choroidal malperfusion, while those with a shorter disease duration did not.

**Conclusion:**

OCTA analysis confirmed impairment of retinal and choroidal microperfusion in SSc patients, supporting the hypothesis of wide spreading vascular injury. In early stages, either the retinal or the choroidal perfusion seems to be involved, while later on, vascular impairment affects both tissues alike. Both, retinal and choroidal examinations should be considered as soon as the diagnosis of SSc is made, to avoid missing out on early alterations.

## Background

Systemic sclerosis (SSc) is a chronic multisystem connective tissue disease, which is characterized by microvascular dysfunction, chronic inflammation, and generalized fibrosis. The vascular involvement is not just limited to the peripheral microcirculation of the skin but is also observed in other organs, such as the eyes [1]. Fibroproliferative vasculopathy in SSc is thought to result from a primary endothelial cell dysfunction with disruption of the endothelium, mononuclear cell infiltration of the vessel wall, and prolonged vasospasm, resulting in a hypoxic effect with tissue damage and atrophy [2, 3].

Numerous ophthalmic manifestations have been reported in SSc patients, involving both anterior and posterior segments [4]. The retinal tissue has the highest level of oxygen extraction per blood volume, while the choroidal vessels have the highest blood flow in the human body [5, 6]. Since the retinal and choroidal microvasculature is particularly susceptible to systemic vascular changes, as observed in diseases such as hypertension and diabetes mellitus, it would be ideal for monitoring disease progression in SSc patients. Additionally, thicknesses of the choroid and retina have been suggested as potential inflammatory biomarkers for autoimmune diseases with a vascular component [7–9].

So far, choroidal and retinal abnormalities, such as those reported in SSc patients, have been evaluated by optical coherence tomography (OCT) and fluorescence angiography (FA) [10–12]. However, OCT, on the one hand, cannot assess the perfusion state because the acquired images only offer morphological information and FA, on the other hand, is a quite laborious, invasive, long-lasting procedure, which needs an experienced ophthalmologist to review the images. With the recent development of optical coherence tomography angiography (OCTA), the vascular network of the retina, as well as the choroid, is assessable from a functional and dynamic point of view. OCTA generates slab-segmented, high-quality, angiographic images in real-time and in a non-invasive setting [13].

Our study group previously suggested reduced perfusion of the retina in patients with SSc by using OCTA [14]. Likewise, we found significant impairment of all choroidal sublayers in SSc patients compared to healthy subjects [15]. Therefore, we decided to review the data with an increased sample size and focused on possible interactions between retinal and choroidal impairment in SSc.

## Methods

This age- and sex-matched, case–control study was approved by the institutional review board at the University of Lübeck (vote reference number 17–008) and was conducted in accordance with the Declaration of Helsinki. All participants received detailed information about the study and written informed consent was obtained from each subject before enrolment. Ethnically all participants were Caucasian and they underwent a thorough examination including mean arterial pressure (MAP), refraction, best-corrected visual acuity (BCVA) in Snellen, intraocular pressure (IOP), axial length (AL), slit-lamp biomicroscopy, and macular enhanced-depth imaging (EDI) OCT as well as OCTA. The maximum permissible spherical and cylindrical aberration was ± 3 and ± 1 diopters, respectively. Only individuals with a BCVA of at least 20/25 and normal ocular findings were included in this study. SSc patients also underwent rheumatological examinations, including autoantibody profiles and evaluation of the capillaroscopic skin ulcer risk index (CSURI), a tool to quantify the microangiopathy using nail fold videocapillaroscopy, as well as the modified Rodnan skin score (MRSS), an established measure of skin thickness in SSc [16, 17].

Imaging was performed without prior pupil dilatation using the HS-100 (Canon, Tokyo, Japan) OCT/OCTA device. To avoid physiological diurnal variations of ocular perfusion, all images were captured around noon and were also taken by a single, trained operator [18, 19]. Each imaging session included EDI-OCT scans (10 × 10 mm^2^), for a more detailed view of the choroid, and OCTA (3 × 3 mm^2^) volumetric scans of the posterior pole. Only images of high quality (signal strength ≥ 7), centered on the fovea, without motion, segmentation and projection artifacts were accepted to guarantee reliable analysis [20].

Following previously published protocols, subfoveal choroidal thickness (SFCT), as well as the thickness of the choroidal vascular sublayers, such as choriocapillaris (CC), Sattler’s layer (SL), and Haller’s layer (HL), were measured manually in the EDI-OCT scans [15, 21]. Manual measurements were performed by two experienced graders who were blinded to the clinical information of the examined eyes. Macular volume (MV) was acquired according to the Early Treatment Diabetic Retinopathy Study (ETDRS) grid [22]. Choroidal OCTA data were manually segmented in all B-scans to get 20 µm slabs of each choroidal sublayer, according to previously published protocols [18, 23]. The retinal angiograms were segmented according to the manufacturer’s default setting to produce en face images of the superficial capillary plexus, the deep capillary plexus, as well as a full retina slab. Each angiogram was exported into ImageJ (NIH, Version 1.48b, Bethesda, USA) and binarized by the Otsu method, which is an automatic threshold selection from grey-level histograms, to determine the percentage of white and black pixels [24]. Superficial retinal perfusion (SRP), deep retinal perfusion (DRP) and full retinal perfusion (FRP), as well as CC perfusion (CCP), were calculated by scoring the percentage of white pixels in relation to the number of total pixels, while for SL perfusion (SLP) and HL perfusion (HLP) the percentage of black pixels was taken into account [14, 19, 25].

Exploratory data analysis was performed using IBM SPSS (Version 24.0, Armonk, NY, USA) and Prism GraphPad (Version 8.0, La Jolla, CA, USA). BCVA measurements in decimal Snellen were converted to the logarithm of the minimum angle of resolution (logMAR). Metrics of both eyes were averaged for each subject. The Shapiro–Wilk test was used to check for normality of all obtained data, and the Mann–Whitney U test was used to compare between groups. The interaction of OCTA perfusion and various epidemiological as well as clinical parameters were evaluated by Spearman’s rank correlation for the whole cohort and repeated after subgroup allocation based on disease duration. For all tests, values of p < 0.05 were considered statistically significant.

## Results

Thirty eyes of 15 SSc patients without clinical evidence of ocular involvement and 30 eyes of 15 age- and gender-matched healthy controls were recruited. Table 1 reports demographic and clinical characteristics of the enrolled patients. The median age of all participants was 63 (49–77) years, and the median disease duration in SSc patients was 60 (13–228) months. Baseline parameters with potential impact on the OCT and OCTA results, such as BCVA (p = 0.748), IOP (p = 0.195), AL (p = 0.783), and MAP (p = 0.365), did not show significant differences between groups.

**Table 1:** Demographic and clinical characteristics of enrolled systemic sclerosis patients

Parameter	Distribution or median (min–max)
Gender (female/male)	11/4
Age (years)	63 (49–77)
Disease duration (months)	60 (13–228)
Classification (limited/diffuse cutaneous SSc)	12/3
Vasodilatative comedication (*Calcium channel blockers; Phosphodiesterase type 5 inhibitor inhibitors; Endothelin receptor antagonist; Prostacyclin analogue*)	13/15
Immunosuppressive comedication (*Methotrexate; Mycophenolate mofetil; Azathioprine; Hydroxychloroquine*)	12/15
Modified Rodnan Skin Score (0–51)	6 (2–24)
Raynaud +	15/15
History of digital ulcers	12/15
Capillaroscopic skin ulcer risk Index	0.6 (0–41.7)
Arthritis	8/15
Pulmonary fibrosis	9/15
Erythrocyte sedimentation rate (mm)	18 (12–60)
C-Reactive protein (mg/l)	5.1 (0.9–16.6)
ANA +	15/15
Anti-RNA polymerase III +	3/15
Anti-centromere +	7/15
Anti-Scl70 +	6/15

**Fig. 1:**
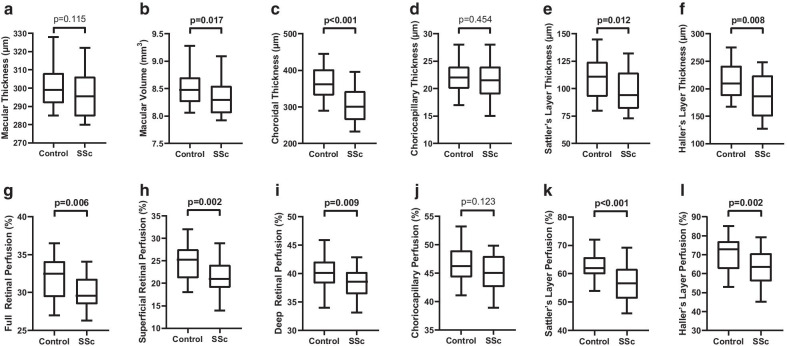
Macular thickness (**a**), macular volume (**b**), choroidal thickness (**c**), choriocapillary thickness (**d**), Sattler’s layer thickness (**e**), Haller’s layer thickness (**f**), full retinal perfusion (**g**), superficial retinal perfusion (**h**), deep retinal perfusion (**i**), choriocapillary perfusion (**j**), Sattler’s layer perfusion (**k**), as well as Haller’s layer perfusion (**l**) were compared between patients with systemic sclerosis and matched controls. Data are presented as box and whisker plots with median, lower as well as upper extreme. Significant differences were tested using Mann–Whitney-U test. P < 0.05 was considered statistically significant

The central macular thickness (CMT) did not differ significantly between SSc patients and healthy controls (p = 0.115) (Fig. 1a). However, SSc patients showed a reduced MV (p = 0.017, Fig. 1b). Thickness analyzation of the choroid revealed a significantly thinner choroid in SSc patients (p < 0.001) compared to healthy controls (Fig. 1c). Considering the choroidal substructures, only SL (p = 0.012) and HL (p = 0.008) were thinner in patients with SSc, while the thickness of the CC (p = 0.454) was similar in both groups (Fig. 1d–f). The FRP was significantly lower in SSc patients (p = 0.006) and substructure analysis revealed that both retinal plexus, the SRP (p = 0.002) and the DRP (p = 0.009), were involved (Fig. 1g–i). The choroidal sublayer perfusion showed no statistically significant difference between SSc patients and healthy controls for the CC (p = 0.123), however, the SL (p < 0.001) and HL perfusion (p = 0.002) were significantly reduced in SSc patients (Fig. 1j–l).

In patients with SSc, HLT (ρ = − 0.461; p = 0.01), SLP (ρ = − 0.381; p = 0.045), and HLP (ρ = − 0.451; p = 0.033) significantly correlated with the patients’ age. Further more, we found statistically significant correlation between SLT and MRSS (ρ = − 0.365; p = 0.047) as well as HLP and CSURI (ρ = − 0.406; p = 0.026), as shown in Table 2.

**Table 2:** Correlation analyses of all systemic sclerosis patients

Parameter	Age	DD	MRSS	CSURI
*CMT*				
CC	− 0.232	− 0.013	− 0.042	− 0.164
P	0.217	0.949	0.824	0.386
*CMV*				
CC	− 0.141	− 0.072	− 0.090	− 0.217
P	0.994	0.706	0.637	0.250
*CT*				
CC	− 0.277	− 0.012	− 0.007	− 0.183
P	0.138	0.949	0.971	0.332
*CCT*				
CC	− 0.100	− 0.042	− 0.199	− 0.052
P	0.601	0.825	0.293	0.787
*SLT*				
CC	− 0.343	− 0.156	− 0.365	− 0.354
P	0.064	0.412	*0.047*	0.055
*HLT*				
CC	− 0.461	− 0.004	− 0.138	− 0.126
P	*0.010*	0.983	0.468	0.506
*FRP*				
CC	− 0.239	− 0.018	− 0.027	− 0.148
P	0.204	0.925	0.886	0.434
*SRP*				
CC	− 0.087	− 0.155	− 0.255	− 0.201
P	0.648	0.414	0.174	0.286
*DRP*				
CC	− 0.259	− 0.032	− 0.188	− 0.054
P	0.286	0.867	0.320	0.778
*CCP*				
CC	− 0.201	− 0.076	− 0.213	− 0.076
P	0.288	0.704	0.259	0.690
*SLP*				
CC	− 0.381	− 0.083	− 0.064	− 0.007
P	*0.045*	0.664	0.736	0.971
*HLP*				
CC	− 0.451	− 0.150	− 0.144	− 0.406
P	*0.033*	0.429	0.447	*0.026*

Italic values are statistically significant (P < 0.05)

*CC* Correlation Coefficient, *CCP* Choriocapillaris Perfusion, *CCT* Choriocapillaris Thickness, *CMT* Central Macular Thickness, *CMV* Central Macular Volume, *CSURI* Capillaroscopic skin Ulcer Risk Index, *CT* Choroidal Thickness, *DD* Disease Duration, *DRP* Deep Retinal Perfusion, *FRP* Full Retinal Perfusion, *MRSS* Modified Rodnan Skin Score, *HLP* Haller’s Layer Perfusion, *HLT*, Haller’s Layer Thickness, *SLP* Sattler’s Layer Perfusion, *SLT* Sattler’s Layer Thickness, *SRP* Superficial Retinal Perfusion

Table 3 reports the interplay between retinal and choroidal malperfusion in SSc patients. FRP correlated positively with SRP (ρ = 0.429; p = 0.021) and DRP (ρ = 0.641; p = 0.003), as well as SRP and DRP with each other (ρ = 0.438; p = 0.015). Choroidal substructure analysis revealed significant correlation between SLP and HLP (ρ = 0.672; p < 0.001). However, the perfusion values in none of the retinal slabs showed a statistically significant correlation with the perfusion in any choroidal sublayer.

**Table 3:** Correlation analyses of all systemic sclerosis patients

Parameter	FRP	SRP	DRP	CCP	SLP	HLP
*FRP*						
CC	1	0.429	0.641	0.019	0.028	0.122
P		*0.021*	*0.003*	0.919	0.881	0.520
*SRP*						
CC	0.429	1	0.438	0.011	0.075	0.004
P	*0.021*		*0.015*	0.954	0.692	0.981
*DRP*						
CC	0.641	0.438	1	− 0.166	0.117	0.084
P	*0.003*	*0.015*		0.380	0.538	0.659
*CCP*						
CC	0.019	0.011	0.166	1	0.224	0.257
P	0.919	0.954	0.380		0.324	0.308
*SLP*						
CC	0.028	0.075	0.117	0.224	1	0.672
P	0.881	0.692	0.538	0.324		*< 0.001*
*HLP*						
CC	0.122	0.004	0.084	0.257	0.672	1
P	0.520	0.981	0.659	0.308	*< 0.001*	

Italic values are statistically significant (P < 0.05)

*CC* Correlation Coefficient, *CCP* Choriocapillaris Perfusion, *CCT* Choriocapillaris Thickness, *CMT* Central Macular Thickness, *CMV* Central Macular Volume, *CSURI* Capillaroscopic skin Ulcer Risk Index, *CT* Choroidal Thickness, *DRP* Deep Retinal Perfusion, *FRP* Full Retinal Perfusion, *MRSS* Modified Rodnan Skin Score, *HLP* Haller’s Layer Perfusion, *HLT* Haller’s Layer Thickness, *SLP* Sattler’s Layer Perfusion, *SLT* Sattler’s Layer Thickness, *SRP* Superficial Retinal Perfusion; P < 0.05 was considered statistically significant

When analyzing the SSc subgroup with a disease duration of less than 60 months, correlation values did not differ significantly from the overall group, and still no significant correlation was found between retinal and choroidal perfusion (Table 4). However, in subjects with a disease duration of more than 60 months a significant correlation between perfusion values of the retinal and the choroidal sublayers were found (Table 5). FRP significant correlated with HLP (ρ = 0.429; p = 0.039), while DRP correlated with both, SLP (ρ = 0.427; p = 0.039) and HLP (ρ = 0.464; p = 0.01).

**Table 4:** Correlation analyses of all systemic sclerosis patients with disease duration < 60 months (n = 12 eyes)

Parameter	FRP	SRP	DRP	CCP	SLP	HLP
*FRP*						
CC	1	0.496	0.685	0.015	0.078	0.101
P		*0.012*	*< 0.001*	0.933	0.752	0.481
*SRP*						
CC	0.496	1	0.555	0.041	0.114	0.085
P	*0.012*		*0.007*	0.914	0.564	0.716
*DRP*						
CC	0.685	0.555	1	0.184	0.152	0.116
P	*< 0.001*	*0.007*		0.445	0.488	0.545
*CCP*						
CC	0.015	0.041	0.184	1	0.305	0.371
P	0.933	0.914	0.445		0.180	0.093
*SLP*						
CC	0.078	0.114	0.152	0.305	1	0.745
P	0.752	0.564	0.488	0.180		*< 0.001*
*HLP*						
CC	0.101	0.085	0.116	0.371	0.745	1
P	0.481	0.716	0.545	0.093	*< 0.001*	

Italic values are statistically significant (P < 0.05)

*CC* Correlation Coefficient, *CCP* Choriocapillaris Perfusion, *CCT* Choriocapillaris Thickness, *CMT* Central Macular Thickness, *CMV* Central Macular Volume, *CSURI* Capillaroscopic skin Ulcer Risk Index, *CT* Choroidal Thickness, *DRP* Deep Retinal Perfusion, *FRP* Full Retinal Perfusion, *MRSS* Modified Rodnan Skin Score, *HLP* Haller’s Layer Perfusion, *HLT* Haller’s Layer Thickness, *SLP* Sattler’s Layer Perfusion, *SLT* Sattler’s Layer Thickness, *SRP* Superficial Retinal Perfusion; P < 0.05 was considered statistically significant

**Table 5:** Correlation analyses of all systemic sclerosis patients with disease duration > 60 months (n = 16 eyes)

Parameter	FRP	SRP	DRP	CCP	SLP	HLP
*FRP*						
CC	1	0.402	0.558	0.344	0.395	0.429
P		*0.045*	*0.006*	0.151	0.064	*0.039*
*SRP*						
CC	0.402	1	0.517	0.274	0.341	0.385
P	*0.045*		*0.010*	0.289	0.162	0.071
*DRP*						
CC	0.558	0.517	1	0.389	0.427	0.464
P	*0.006*	*0.010*		0.066	*0.039*	*0.010*
*CCP*						
CC	0.344	0.274	0.389	1	0.385	0.397
P	0.151	0.289	0.066		0.069	0.055
*SLP*						
CC	0.395	0.341	0.427	0.385	1	0.664
P	0.064	0.162	*0.039*	0.069		*< 0.001*
*HLP*						
CC	0.429	0.385	0.464	0.397	0.664	1
P	*0.039*	0.071	*0.010*	0.055	*< 0.001*	

Italic values are statistically significant (P < 0.05)

*CC* Correlation Coefficient, *CCP* Choriocapillaris Perfusion, *CCT* Choriocapillaris Thickness, *CMT* Central Macular Thickness, *CMV* Central Macular Volume, *CSURI* Capillaroscopic skin Ulcer Risk Index, *CT* Choroidal Thickness, *DRP* Deep Retinal Perfusion, *FRP* Full Retinal Perfusion, *MRSS* Modified Rodnan Skin Score, *HLP* Haller’s Layer Perfusion, *HLT* Haller’s Layer Thickness, *SLP* Sattler’s Layer Perfusion, *SLT* Sattler’s Layer Thickness, *SRP* Superficial Retinal Perfusion; P < 0.05 was considered statistically significant

## Discussion

The present study confirms and expands the evidence for retinal and choroidal involvement in patients with SSc by using OCTA. Asymptomatic eyes of patients with SSc showed reduced retinal as well as choroidal sublayer perfusion compared to healthy controls. For the first time, we were able to demonstrate that in the early stages of SSc, there was no correlation between the vascular involvement of the retina and choroid. However, as disease duration progressed the reduced perfusion of the retina and choroid correlated significantly. We were able to confirm previous findings of a lower SRP, DRP, and FRP, as well as SLP and HLP, in a larger-scaled cohort of SSc patients [14, 15].

CT is mainly determined by the thickness of SL and HL, which mostly consist of medium to large caliber vessels. The extravascular stroma makes up only a small part of the overall CT. Since alterations in choroidal thickness reflect the status of the vasculature within, it has been used as an indirect index of choroidal perfusion [26]. We were able to confirm this theory by demonstrating a significantly reduced SLP and HLP but not CCP in SSc patients, according to the results of the thickness measurements.

Besides us, only Kılınç Hekimsoy et al. investigated the retinal and choroidal perfusion in SSc patients by using OCTA [27]. We were able to confirm their findings of a reduced SRP and DRP perfusion in SSc. Regarding choroidal metrics, they did not find significant differences in CCP as well as CC thickness. However, they did not investigate choroidal substructures. Regarding changes of CMT due to SSc, we did not find significant differences between groups. While Kılınç Hekimsoy et al. reported similar results, Esen et al. found a thinner CMT in SSc patients compared to healthy subjects. A possible explanation could be the younger age of the control group, compared to our study, knowing that retinal thickness decreases with age [28]. However, we were able to demonstrate reduced MV in patients with SSc.

One aspect that has not yet been discussed in the literature is the different involvement of the choroidal and retinal vasculature in SSc patients, depending on the disease duration. The pathology of SSc is characterized by a chronic and self-amplifying multifocal process with immune activation and vascular injury, leading to tissue fibrosis and vascular obstruction, mainly targeting small arteries and capillaries [29]. Microvascular injury and endothelial cell activation, leading to vascular damage are the earliest alterations in SSc [30]. The progressive vascular damage causes rarefication in capillaries, vessel wall thickening due to smooth muscle and intimal proliferation with luminal narrowing, which lead to tissue ischemia. A conspicuous feature of SSc is its variability between patients and heterogeneity in clinical manifestations, as well as its tempo of disease progression. However, data proposes that patients with early-stage diseases show high prevalence of digital ulcers, gastrointestinal alterations, and echocardiogram abnormalities [31]. Severe complications of the kidneys, heart, lungs, and the gastrointestinal tract usually develop within 3 years of disease onset [32]. Several studies tried to identify predictive factors, such as patients’ age, presence of digital ulcer, lung fibrosis, and CRP elevation, that are associated with a fast disease progression and lead to organ failure [33, 34]. However, it is still challenging to predict disease progression in SSc patients. It is not yet known, at what stage of the disease retinal or choroidal involvement occurs. Evidence suggests that diffuse alterations in the microcirculation may already be present in the earliest phases of the disease, even before the onset of any clinical signs [3]. However, our general data does not reveal any significant correlation between the vascular malperfusion of the retina and choroid. Likewise, the subgroup analysis of patients with disease duration of less than 60 months did not reveal any significant correlation between the retinal and choroidal perfusion, suggesting that either the retinal or the choroidal vasculature is involved in the pathology. However, perfusion values of the retina and the choroid positively correlated in SSc patients with a disease duration of more than 60 months. One could conclude that in the early stages of the disease, either the retina or choroid is affected by the vasculopathy, and with increasing disease duration, both tissues become affected.

Explanatory approaches for the different involvement of both tissues are their dissimilar vascular regulatory mechanisms. While histological studies have revealed a rich supply of autonomic vasoactive innervation for the choroid, the nerves do not go further into the retina [35]. Therefore, retinal blood flow is mainly under autoregulation by both myogenic and local metabolic mechanisms [6]. Besides the fact of missing autonomic innervation, the retina features an immune privileged microvascular environment with absence of resident fibroblasts [36]. Since a significant proportion of vascular disease in SSc is thought to result from increased migration of activated resident fibroblasts into the vessel wall, leading to augmented extracellular matrix deposition with vascular stiffness and dysfunction, the characteristics of the retina may provide sparing from this process [37]. Furthermore, systemic hypertension is a factor that is hypothesized to influence vascular structures, especially in patients with advanced SSc and renal involvement. Due to the absence of neuronal innervation in retinal vascular beds, in contrast to the choroid, changes in systemic perfusion pressure have only a negligible influence on retinal blood flow [38]. Though, the choroidopathy could result from concomitant systemic hypertension due to the poor autoregulatory mechanisms and from SSc itself.

Limitations of the present study, which must be taken into account when interpreting the results, are the relatively small number of patients, leading to a mainly exploratory data analysis, and the restriction by using only a single OCTA device. Exploratory data analysis is an important tool to extract novel information from a data-set. It allows us to single out new directions of future research, since very little is known about retinal and choroidal microvascular impairment in SSc patients to date. However, these exploratory results should always be considered with caution and confirmative studies on a larger study population enabling adjustments for multiple comparisons are mandatory to validate the results. As OCTA is a tool for quantification of the retinal and choroidal vasculature, it is not suitable to determine the speed of the blood circulation. Therefore, we may have missed out on significant changes of velocity for example due to autoregulatory mechanisms. Furthermore, the scanned area of 3 × 3 mm^2^ only represents a small part of the retina and the choroid and may have been too small to suggest a global impairment of the ocular perfusion in SSc. Beyond, the understanding and interpretation of various signals in the choroidal vasculature on OCTA analysis are discussed controversy and need further research. Nevertheless, due to the rarity of the disease the number of included patients is still meaningful and we obtained statistically significant results. So far, ophthalmological examinations of SSc patients focus just on retinal abnormalities. However, based on our study results, we suggest that both, retinal and choroidal examination, should be considered as soon as the diagnosis of SSc is made. However, diagnostic characteristics such as sensitivity, specificity as well as the predictive and clinical value of OCTA as a potential biomarker for SSc need to be evaluated in further studies.

## Conclusion

OCTA is able to detect microvascular impairment of retinal as well as choroidal vessels in patients with SSc. Based on the study results, in early stages of SSc either the retinal or the choroidal perfusion seems to be reduced, independently. As disease progresses, both tissues get involved in the process of global vascular damage. Therefore, it should be considered whether in addition to the examination of the retinal vessels, also the choroidal vasculature needs to be evaluated to avoid missing out on early microvascular alterations of the disease. The detection of ocular microvascular alterations could identify patients with early disease progressions and influence treatment decisions. However, further studies with confirmatory data analysis on a larger number of subjects will be necessary to corroborate our findings.

## Data Availability

The datasets used and analyzed during the current study are available from the corresponding author on reasonable request.

## Abbreviations

AL: Axial length
BCVA: Best-corrected visual acuity
CC: Choriocapillaris
CCP: Choriocapillaris perfusion
CCT: Choriocapillaris thickness
CMT: Central macular thickness
CMV: Central macular volume
CSURI: Capillaroscopic skin ulcer risk index
CT: Choroidal thickness
DD: Disease duration
DRP: Deep retinal perfusion
EDI-OCT: Enhanced-depth imaging optical coherence tomography
FA: Fluorescence angiography
FRP: Full retinal perfusion
HL: Haller’s layer
HLP: Haller’s layer perfusion
HLT: Haller’s layer thickness
IOP: Intraocular pressure
MAP: Mean arterial pressure
MRSS: Modified Rodnan skin score
OCT: Optical coherence tomography
OCTA: Optical coherence tomography angiography
SFCT: Subfoveal choroidal thickness
SL: Sattler’s layer
SLP: Sattler’s layer perfusion
SLT: Sattler’s layer thickness
SRT: Superficial retinal perfusion
SSc: Systemic sclerosis

## References

[1] Allanore Y, Simms R, Distler O, Trojanowska M, Pope J, Denton CP (2015). Systemic sclerosis. Nat Rev Dis Primers.

[2] Flavahan NA, Flavahan S, Mitra S, Chotani MA (2003). The vasculopathy of Raynaud’s phenomenon and scleroderma. Rheum Dis Clin N Am..

[3] Matucci-Cerinic M, Kahaleh B, Wigley FM (2013). Review: evidence that systemic sclerosis is a vascular disease. Arthritis Rheum.

[4] de Gomes AFB, Santhiago MR, Magalhães P, Kara-Junior N, de Azevedo MNL, Moraes HV (2011). Ocular findings in patients with systemic sclerosis. Clinics.

[5] Bill A (1975). Blood circulation and fluid dynamics in the eye. Physiol Rev.

[6] Luo X, Shen Y-M, Jiang M-N, Lou X-F, Shen Y (2015). Ocular blood flow autoregulation mechanisms and methods. J Ophthalmol.

[7] Ferreira CS, Beato J, Falcão MS, Brandão E, Falcão-Reis F, Carneiro ÂM (2017). Choroidal thickness in multisystemic autoimmune diseases without ophthalmologic manifestations. Retina.

[8] Karadag AS, Bilgin B, Soylu MB (2017). Comparison of optical coherence tomographic findings between Behcet disease patients with and without ocular involvement and healthy subjects. Arq Bras Oftalmol.

[9] Steiner M, Esteban-Ortega MDM, Muñoz-Fernández S (2019). Choroidal and retinal thickness in systemic autoimmune and inflammatory diseases: a review. Surv Ophthalmol.

[10] Esen E, Tas DA, Sizmaz S, Turk I, Unal I, Demircan N (2017). Evaluating choroidal characteristics in systemic sclerosis using enhanced depth imaging optical coherence tomography. Ocul Immunol Inflamm.

[11] Ingegnoli F, Gualtierotti R, Pierro L, Del Turco C, Miserocchi E, Schioppo T (2015). Choroidal impairment and macular thinning in patients with systemic sclerosis: the acute study. Microvasc Res.

[12] Waszczykowska A, Goś R, Waszczykowska E, Dziankowska-Bartkowiak B, Jurowski P (2013). Prevalence of ocular manifestations in systemic sclerosis patients. Arch Med Sci.

[13] Rommel F, Siegfried F, Kurz M, Brinkmann MP, Rothe M, Rudolf M (2018). Impact of correct anatomical slab segmentation on foveal avascular zone measurements by optical coherence tomography angiography in healthy adults. J Curr Ophthalmol.

[14] Rothe M, Rommel F, Klapa S, Humrich JY, Nieberding R, Lange T (2019). Evaluation of retinal microvascular perfusion in systemic sclerosis: a case–control study. Ann Rheum Dis.

[15] Ranjbar M, Rothe M, Klapa S, Lange T, Prasuhn M, Grisanti S (2019). Evaluation of choroidal substructure perfusion in patients affected by systemic sclerosis: an optical coherence tomography angiography study. Scand J Rheumatol..

[16] Sebastiani M, Manfredi A, Colaci M, D’amico R, Malagoli V, Giuggioli D (2009). Capillaroscopic skin ulcer risk index: a new prognostic tool for digital skin ulcer development in systemic sclerosis patients. Arthritis Rheum..

[17] Valentini G, D’Angelo S, Della Rossa A, Bencivelli W, Bombardieri S (2003). European Scleroderma Study Group to define disease activity criteria for systemic sclerosis. IV. Assessment of skin thickening by modified Rodnan skin score. Ann Rheum Dis..

[18] Siegfried F, Rommel F, Rothe M, Brinkmann MP, Sochurek JAM, Freitag J (2019). Evaluating diurnal changes in choroidal sublayer perfusion using optical coherence tomography angiography. Acta Ophthalmol.

[19] Rommel F, Siegfried F, Sochurek JAM, Rothe M, Brinkmann MP, Kurz M (2019). Mapping diurnal variations in choroidal sublayer perfusion in patients with idiopathic epiretinal membrane: an optical coherence tomography angiography study. Int J Retina Vitreous.

[20] Lauermann JL, Woetzel AK, Treder M, Alnawaiseh M, Clemens CR, Eter N (2018). Prevalences of segmentation errors and motion artifacts in OCT-angiography differ among retinal diseases. Graefes Arch Clin Exp Ophthalmol.

[21] Ranjbar M, Prasuhn M, Kurz M, Holzhey A, Rommel F, Brinkmann MP (2019). Subfoveal choriocapillaris, Sattler’s and Haller’s layer thickness predict clinical response to stereotactic radiotherapy in neovascular age-related macular degeneration patients. J Curr Ophthalmol.

[22] Grading diabetic retinopathy from stereoscopic color fundus photographs—an extension of the modified Airlie House classification. ETDRS report number 10. Early Treatment Diabetic Retinopathy Study Research Group. Ophthalmology. 1991;98(5 Suppl):786–806. 2062513

[23] Gabriel M, Esmaeelpour M, Shams-Mafi F, Hermann B, Zabihian B, Drexler W (2017). Mapping diurnal changes in choroidal, Haller’s and Sattler’s layer thickness using 3-dimensional 1060-nm optical coherence tomography. Graefes Arch Clin Exp Ophthalmol.

[24] Otsu N (1979). A threshold selection method from gray-level histograms. IEEE Trans Syst Man Cybern.

[25] Nicolò M, Rosa R, Musetti D, Musolino M, Saccheggiani M, Traverso CE (2017). Choroidal vascular flow area in central serous chorioretinopathy using swept-source optical coherence tomography angiography. Invest Ophthalmol Vis Sci.

[26] Kim M, Kim SS, Kwon HJ, Koh HJ, Lee SC (2012). Association between choroidal thickness and ocular perfusion pressure in young, healthy subjects: enhanced depth imaging optical coherence tomography study. Invest Ophthalmol Vis Sci.

[27] Kılınç Hekimsoy H, Şekeroğlu MA, Koçer AM, Akdoğan A (2019). Analysis of retinal and choroidal microvasculature in systemic sclerosis: an optical coherence tomography angiography study. Eye (Lond)..

[28] Alamouti B, Funk J (2003). Retinal thickness decreases with age: an OCT study. Br J Ophthalmol.

[29] Rabquer BJ, Koch AE (2012). Angiogenesis and vasculopathy in systemic sclerosis: evolving concepts. Curr Rheumatol Rep.

[30] Trojanowska M (2010). Cellular and molecular aspects of vascular dysfunction in systemic sclerosis. Nat Rev Rheumatol.

[31] Valentini G, Cuomo G, Abignano G, Petrillo A, Vettori S, Capasso A (2011). Early systemic sclerosis: assessment of clinical and pre-clinical organ involvement in patients with different disease features. Rheumatology (Oxford).

[32] Steen VD, Medsger TA (2000). Severe organ involvement in systemic sclerosis with diffuse scleroderma. Arthritis Rheum.

[33] Maurer B, Graf N, Michel BA, Müller-Ladner U, Czirják L, Denton CP (2015). Prediction of worsening of skin fibrosis in patients with diffuse cutaneous systemic sclerosis using the EUSTAR database. Ann Rheum Dis.

[34] Becker M, Graf N, Sauter R, Allanore Y, Curram J, Denton CP (2019). Predictors of disease worsening defined by progression of organ damage in diffuse systemic sclerosis: a European Scleroderma Trials and Research (EUSTAR) analysis. Ann Rheum Dis.

[35] Laties AM (1967). Central retinal artery innervation. Absence of adrenergic innervation to the intraocular branches. Arch Ophthalmol..

[36] Aissopou EK, Bournia V-K, Protogerou AD, Panopoulos S, Papaioannou TG, Vlachoyiannopoulos PG (2015). Intact calibers of retinal vessels in patients with systemic sclerosis. J Rheumatol.

[37] Gilbane AJ, Denton CP, Holmes AM (2013). Scleroderma pathogenesis: a pivotal role for fibroblasts as effector cells. Arthritis Res Ther.

[38] Rassam SMB, Patel V, Chen HC, Kohner EM (1996). Regional retinal blood flow and vascular autoregulation. Eye.

